# Assessment of infraorbital foramen position using computed tomography-scan in a cohort of Cameroonian adults: landmarks in facial surgery and anesthesiology

**DOI:** 10.11604/pamj.2023.45.134.37733

**Published:** 2023-07-19

**Authors:** Akaba Désiré, Messina Ebogo, Mballa Amougou, Ntcham Essono, Ongolo Zogo

**Affiliations:** 1 Department of Morphologic Sciences, Clinical Anatomy, Faculty of Medicine and Biomedical Sciences, University of Yaoundé I, Yaoundé, Cameroon,; 2 Department of Oral and Maxillofacial Surgery, Cheikh Anta Diop University of Dakar, Dakar, Senegal,; 3 Department of Radiology and Medical Imaging, Faculty of Medicine and Biomedical Sciences, University of Yaoundé I, Yaoundé, Cameroon

**Keywords:** Infraorbital foramen, morphometry, location, clinical landmarks

## Abstract

**Introduction:**

the infraorbital foramen (IOF) is a hole located in the maxillary bone and delivering passage to the infra orbital vascular-nervous bundle. It is an essential structure in the management of orofacial pathologies. Its precise location allows optimal anesthesia of the infraorbital nerve during cleft lip and palate surgery or alcoholization during the management of essential V2 neuralgia. The aim of our research was to determine the morphology and morphometry of the infraorbital foramen in a sample of the Cameroonian population.

**Methods:**

we included 208 CT-scans of patients meeting our search criteria. We determined the shape of the IOF and evaluated the transverse and vertical diameters of the IOF. We assessed the distance of the IOF from the maxillary alveolar crest and the infraorbital margin. The Student test was used to determine the association between different variables. The P-value of 0.05 was considered significant and the confidence interval was 95%.

**Results:**

male subjects represented 52.4% (n=109) of our participants and the mean age of our population was 26 years ± 7.3. The mean transverse diameter of the left IOF was 1.97 mm ± 0.51 while 1.78 mm ± 0.53. The IOF was more often medial to the lateral palpebral commissure-nasal wing line on the left and right (78.8% and 72.6%, respectively). Our sample showed that in 54.6% (n=113) of subjects, the IOF was oval on the left side, whereas on the right side, the IOF was oval in 52.3% (n=109) of patients.

**Conclusion:**

our study showed that to locate the IOF in a Cameroonian individual, one must palpate the vestibular mucosa opposite the maxillary first molar. Then, one must follow the line passing over this tooth, the IOF is located at about 7 mm from the infra-orbital border and 16 mm from the lateral nasal wall. We have shown that the IOF is located medial to the line connecting the nasal wing to the external palpebral commissure.

## Introduction

The infraorbital foramen (IOF) is an opening in the maxillary located below the infraorbital margin and is the outer end of the infraorbital canal. It gives passage to the artery, the vein, and to the infra-orbital nerve [1]. The infraorbital nerve follows the maxillary nerve and originates at the infraorbital foramen. It is a sensory nerve that divides into several branches ensuring the sensitivity of the skin of the upper cheek, the skin of the nose, the upper lip, the vestibular mucosa, and the maxillary teeth [1]. This nervous structure is important in the management of pathologies of the orofacial sphere and implant surgery. Infraorbital nerve block anesthesia is used in the treatment of pharmacologically resistant essential neuralgia of the V2 nerve [2]. The anesthesia of this nerve first requires the location of the foramen through which it emerges on the face. During loco-regional anesthesia by a block of the infra-orbital nerve, the surgeon must locate himself by palpating the lower edge of the orbital floor to identify the infra-orbital foramen, then insert the needle upwards to infiltrate a local anesthetic [3]. A traumatic section of the infraorbital vascular-nervous bundle can occur during facial surgery or dental implantology, thus causing a disturbance in the sensitivity of the face and or more or less significant bleeding in this region [4]. It is therefore important to know the location of the infraorbital foramen (IOF) and the direction of the infraorbital canal for effective nerve block. Additionally, the structures around the infraorbital foramen such as the canine fossa and the tuberosity above the foramen seem to be important in deciding the correct direction of the needle [5].

Several methods are used for the morphological and morphometric study of the infra-orbital foramen. This may involve work on dry adult skulls, by cadaveric dissection and per operative study of the foramen is possible. It is possible to study the morphometry of this foramen by means of medical imaging, in particular cone beam computed tomography (CBCT) and CT, which have a very high specification [6]. Authors have shown many variations in position, shape and even number according to race, sex, and ethnicity [4].

In view of the importance of this orifice in oral and maxillofacial surgery and in anesthesiology, it is necessary to study its anatomy and its possible topographical variations in order to avoid any section of the vascular-nervous bundle in transit in the IOF. This study aimed to study the morphology and morphometry of the IOF in a sample of the Cameroonian population.

## Methods

**Study design and setting:** this was a descriptive cross-sectional study that took place over a period of 18 months from March 2020 to September 2021. The CT images of the face used in our research were obtained at the Radiology and Medical Imaging Department of the Yaoundé Cathedral Medical Center during the period of our study.

**Participants:** for this research, images of subjects meeting the criteria of black skin and melanodic Cameroonian type with a usable CT-scan of the face or skull were included. We retained 208 CT-scans of the face of individuals who met our inclusion criteria.

**Inclusion criteria:** in this study, we included: images of Cameroonian patients with brain or facial CT-scans; patients at least 18 years of age; patients with an image clearly showing the IOF.

**Exclusion criteria:** we excluded from our work: any patient with edentulousness that does not allow IOF dental registration; any patient with an image showing trauma to the jawbone; any CT-scan of a subject who has undergone a maxillary osteotomy.

**Data collection:** for each CT-scan image presenting the selected facial bone, we performed a visual analysis of the three-dimensional reconstructions on the frontal plane, in order to clearly highlight the maxillary bone and have a view of the IOF. When the IOF was identified, using a mouse connected to our computer, we made linear plots according to the previously defined variables. Thanks to the measurement module of the medical imaging analysis software Radi Ant Dicom Viewer 2021.2.

**Figure 1:**
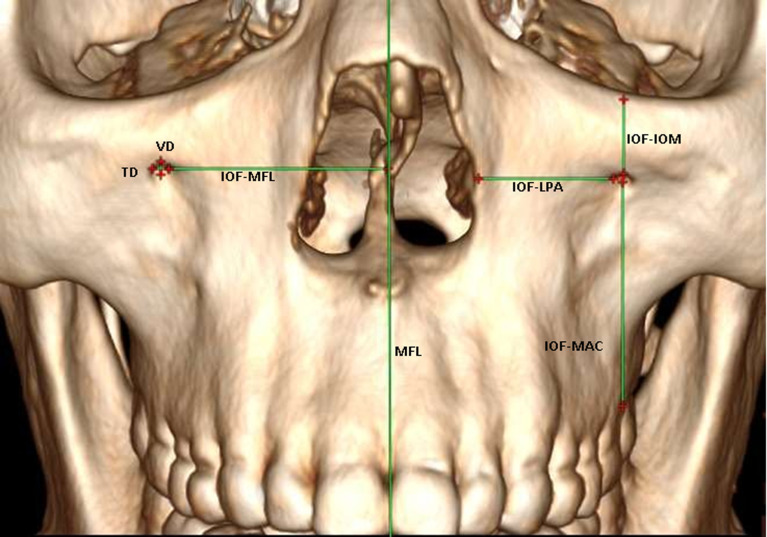
three dimensional representation of a skull showing the different tracings made (VD: vertical diameter; TD: transverse diameter; IOF-MFL: distance infraorbital foramen - median facial line; IOF-IOM: distance infraorbital foramen - lnfraorbital margin; IOF-MAC: distance infraorbital foramen - maxillary alveolar crest; IOF-LPA: distance infraorbital foramen - lateral edge of the piriform aperture; MFL: middle facial line)

**Morphologic investigations:** we evaluated the distances of the different points selected. To do this, we have evaluated the morphometry of different points, in particular: the vertical diameter (VD) of the IOF was determined by drawing a vertical line between the upper and lower edges of the IOF (Figure 1); the transverse diameter (TD) of the IOF was determined by drawing a horizontal line between the medial and lateral edges of the IOF (Figure 1); the shape was considered rounded if the vertical diameter was at the transverse diameter while the foramen was considered oval for a vertical diameter greater than the transverse diameter. Accessory foramen was defined as the presence of an additional foramen on the CT images reviewed.

**Figure 2:**
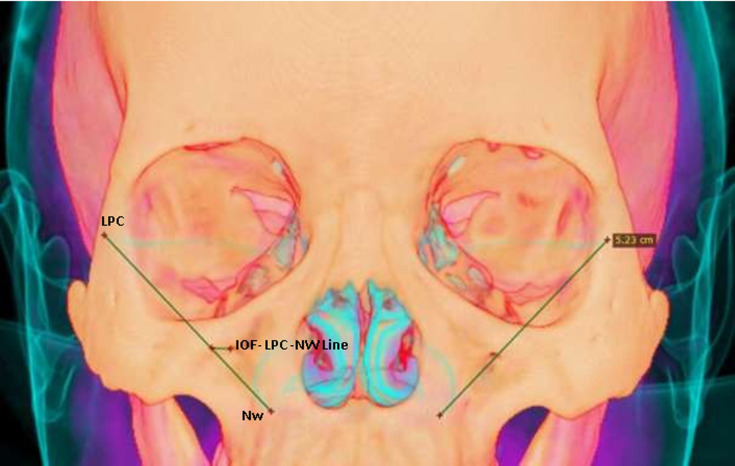
three dimensional superposition of the skin on the skull to obtain mucosal landmarks (IOF-LPC-NW Line: distance lateral palpebral commissure - nasal wing- infraorbital foramen)

The distance between the medial margin of the IOF and the lateral edge of the piriform aperture (LPA) was obtained by measuring the distance between the most medial edge of the foramen and the lateral edge of the piriform aperture (Figure 1). The distance between the lower margin of the IOF and the maxillary alveolar crest (MAC) was determined by measuring the distance between the lower edge of the IOF and the crests of the maxillary teeth, at the level of the alveolar processes of the teeth (Figure 1). The distance foramen-lower orbital wall and infraorbital margin (IOM) were obtained by measuring the distance between the upper edge of the IOF and the lower edge of the orbital wall (Figure 1). The distance between the IOF and the middle facial line (MFL) was determined by measuring the distance between the center of the IOF and the midline (Figure 1). We also determined the position of the IOF with respect to the lateral palpebral commissure-nasal wing (LPC-NW) line (Figure 2). Thus, the distance evaluated in millimeters appeared automatically once the linear plot was made and then was transferred manually to our technical sheet.

**Definitions:** the vertical diameter (VD) was the distance determined by drawing a vertical line between the upper and lower edges of the IOF. The transverse diameter (TD) of the IOF was the distance determined by drawing a horizontal line between the medial and lateral edges of the IOF. The round shape if the vertical diameter was at the transverse diameter while the foramen was considered oval for a vertical diameter greater than the transverse diameter. Accessory foramen was defined as the presence of an additional foramen on the CT images reviewed.

**Statistical analysis:** the information from our data collection technical sheets was recorded in an input mask at the same time as the collection process. Data processing was done by SPSS version 20.1 software based on our variables. We performed the mean and median calculations as an indicator of central tendency and the standard deviation and interquartile range calculations as an indicator of variability with their confidence intervals. We then proceeded to the descriptive analysis of the results. We compared the morphological variables and the morphometric variables according to gender and the side using the Student test. The PË‚0.05 value was taken as statistically significant. We used the Student's t-test for the analysis of inter- and intra-observer reproducibility at the 5% threshold. The P-values found were between 0.23 and 0.57. These values are above the significance threshold, which implies that our study is 95% reproducible.

**Ethical considerations:** the study was conducted in accordance with the guidelines of the Declaration of Helsinki and approved by the Institutional Ethics and Research Committee of the University of Yaoundé I (permit number: 178/UYI/FMSB/VDRC/DAASR/ CSD and approval date of April 16, 2021). The anonymity of the data was respected throughout our research. Permission from the manager of our data collection site was obtained before the start of our investigation.

## Results

**General characteristics of the study population:** we retained 208 CT-scans of the facial mass that met our inclusion criteria and thus represent 416 IOF studied. Male subjects represented 52.4% (n=109) of our participants and the mean age of our population was 26 years ± 7.3.

**Morphology:** our sample showed that in 54.6% (n=113) of subjects, the IOF was oval on the left side, whereas on the right side, the IOF was round in 52.3% (n=109) of patients. An accessory infra-orbital foramen was found in 29.8% (n=62) of subjects in our series on the left, while on the right, the accessory IOF was present in 24.5% (n=51) of individuals. In our sample, in 49.5% (n=103) participants, the left IOF was located on the line passing below the first molar while in 50% (n=104) of the subjects, the right IOF was continuous with the line passing below the first molar (Table 1).

**Table 1:** distribution of infraorbital foramen diameters according to side

Teeth	Left side (%)	Right side (%)
PM1	3 (1.4)	2 (1.01)
PM2	79 (38.0)	79 (38.0)
M1	103 (49.5)	104 (50)
M2	22 (10.6)	23 (11.1)

PM1: first premolar; PM2: second premolar; M1: first molar; M2: second molar

**Morphometry:** the mean transverse diameter of the left IOF was 1.97 mm ± 0.51 while the transverse diameter of the right IOF was 2.10 mm ± 0.52 (Table 2). The mean IOF-IOM (infraorbital margin) distance was 7.17 mm ± 1.91 on the left and 6.87 mm ± 1.87 on the right. We found an IOF-MFL distance of 27.4 mm ± 4.42 on the left and 28.7 mm ± 3.23 on the right (Table 3). Our research showed that the IOF was more often medial to the LPC-NW line on the left and right (78.8% and 72.6%, respectively). The mean distance between the IOF and the LPC-NW line was 5.95 mm ± 2.99 on the left and 5.65 mm ± 3.03 on the right when the IOF was medial to this line (Table 4).

**Table 2:** position of the infraorbital foramen in relation to certain anatomical landmarks according to the side

Variables	Minimum	Means (SD)	Median (CI)	Maximum
**Transverse diameter (mm)**				
Left	0.50	1.97 (0.51)	1.93 [1.60-2.34]	3.39
Right	0.80	2.10 (0.52)	2.08 [1.83-3.62]	3.82
**Vertical diameter (mm)**				
Left	0.44	1.78 (0.53)	1.80 [1.35-2.06]	3.48
Right	0.67	1.69 (0.51)	1.62 [1.32-1.95]	3.63

SD: standard deviation; CI: confidence interval

**Table 3:** position of the infraorbital foramen relative to the teeth

Variables	Minimum	Means (SD)	Median (CI)	Maximum
**IOF - IOM**				
Left	0.63	7.17 (1.91)	7.28 [6.28-8.47]	12.00
Right	1.01	6.87 (1.87)	6.77 [5.92-8.25]	11.10
**IOF - MFL**				
Left	2.82	27.47 (4.42)	27.50 [25.6-30.7]	36.20
Right	3.47	28.72 (3.23)	28.75 [27.0-30.7]	34.70
**IOF - LPA**				
Left	9.26	15.91 (2.70)	16.10 [14.0-17.70]	27.00
Right	2.30	16.40 (2.47)	16.40 [14.2-17.80]	25.60
**IOF - MAC**				
Left	3.00	28.92 (5.31)	29.05 [27.3-31.90]	38.00
Right	2.30	29.72 (4.67)	30.15 [27.3-32.10]	38.10


IOM: infra orbital marginn; MFL: middle facial line; LPA: lateral edge of piriform aperture; MAC: maxillary alveolar crest

**Table 4:** position of the infraorbital foramen in relation to the LPC-NW line

Variables	Frequencies	Percentages (%)	Means (SD)	Median (CI=95%)
**Medial/LPC-NW**				
Left	164	78.8	5,95 (2.99)	5,19 [3.92-6.81]
Right	151	72.6	5.65 (3.03)	5.98 [4.15-7.34]
**Distal/LPC-NW**				
Left	7	3.4	2.49 (0.93)	2,27 [1.32-5.45]
Right	9	4.3	3.18 (2.43)	2,96 [1.40-5.02]
**On the line**				
Left	37	17.8		
Right	48	23.1		

LPC: lateral palpebral commissure; NW: nasal wing; SD: standard deviation; CI: confidence interval

## Discussion

Our sample showed that in 54.6% (n=113) of subjects, the IOF was oval on the left side, whereas, on the right side, the IOF was round in 52.3% (n=109) of patients. An accessory infra-orbital foramen was found in 29.8% (n=62) of subjects in our series on the left, while on the right, the accessory IOF was present in 24.5% (n=51) of individuals. In our sample, 49.5% (n=103) participants, the left IOF was located on the line passing below the first molar. The mean transverse diameter of the left IOF was 1.97 mm ± 0.51 while the transverse diameter of the right IOF was 2.10 mm ± 0.52 (Table 2). The mean IOF-IOM (infraorbital margin) distance was 7.17 mm ± 1.91 on the left and 6.87 mm ± 1.87 on the right. We found an IOF-MFL distance of 27.4 mm ± 4.42 on the left and 28.7 mm ± 3.23 on the right.

The IOF studied in this work were mainly oval in shape representing 54.2% of the sample. Our results are similar to those of Messina Ebogo *et al*. [7], (48% oval-shaped foramen) and are close to the results obtained in the study by Wandee *et al*. [8] which reported 50% oval-shaped foramen. On the other hand, our results are different from those obtained in the study by Veeramuthu *et al*. [9] which reported 29% of oval-shaped foramen.

The IOF was located at the line passing below the first molar (M1). Our result differs from those of Ali Ibrahim *et al*. [10] and Ilayperuma I *et al*. [11] and Zdilla *et al*. [12] who reported a greater presence above the vertical axis of the second premolar (PM2).

The position of the IOF with respect to the line connecting the lateral palpebral commissure to the wing of the nose (LPC-NW) was studied in our research. We found an IOF medial to the LPC-NW line on the left and right respectively in 78.8% and 72.6% of the individuals in our series. The mean distance between the IOF and the LPC-NW line was 5.95 mm ± 2.99 to the left and 5.65 mm ± 3.03 to the right when the IOF was medial to this line. Ercikti N *et al*. [13] in 2006 in a study done on adult cadavers, reported that in 75% of cases, the foramen is located on the LPC-NW line. This is different from the result obtained in our study. This difference could be explained by cephalometric variations between Caucasian and African subjects. Indeed, according to Farkas *et al*. [14], we observe in Africans a hyper facial divergence and a promaxillia, which could modify the position of the IOF.

Our research showed that the average transverse diameter of the left IOF was 1.97 mm ± 0.51 while the transverse diameter of the right IOF was 2.10 mm ± 0.52. The vertical diameter of the IOF was 1.78 mm. Our results are similar to those found by Nneka, *et al*. [15] in Nigeria who reported a transverse diameter of 2.55 mm ± 0.07 mm. Our dimensions are lower than those of Varalakshmi *et al*. [16] and Elias *et al*. [17] in Brazil who respectively reported transverse diameters of 3.76 mm ± 0.85 on the right and 3.90 mm ± 0.96 on the left; 13.31 mm ± 2.19.

The distance between the IOF and the infraorbital wall was 7.17 mm in our sample. This measurement is similar to those of Tewari *et al*. [18] and Ceri *et al*. [19] who found 7.03 mm ± 3.59 and 6.75 mm ± 1.54 respectively. It is lower than the measurement made by Messina Ebogo *et al*. [7] in Senegal who found 10.8 mm ± 0.27. We found an IOF-midline facial distance of 28.72 mm ± 3.23. In the study by Thilakumara *et al*. [20], this distance was 27.73 mm ± 2.43 in Sri Lanka. This result is close to ours, unlike Dagistan S *et al*. [21] who found a result lower than ours, 25.10 mm ± 2.17.

The distance foramen - lateral edge of the piriform aperture (LPA) was 16.40 mm ± 2.47. This result can be superimposed on those of Polo C *et al*. [22] in Switzerland and Ceri *et al*. [19] in Turkey (16.51 mm ± 1.9; 16.61 mm ± 2.64). However, it remains superior to those of Dagistan S *et al*. [21] and Thilakumara *et al*. [20] respectively reported 9.32 mm ± 2.68 in Turkey and 11.96 mm ± 3.45 in Sri Lanka. The distance foramen - crest of the alveolar bone was 29.72 mm ± 4.67. This measure is similar to that of Polo C *et al*. [22] in Switzerland who reported a distance of 30.34 mm ± 3.37. We found an accessory foramen in 29.8% of the individuals in our sample. Our results are lower than those reported by Polo *et al*. [22] in Switzerland (46.7%). Our prevalence of accessory foramen was however higher than that reported by Mugurel *et al*. [23] in Romania in 2020 (10%). These variations could be explained by the racial difference.

The main strength of our study is that it was carried out using CT-scans; this is an excellent means of studying the morphometry of the IOF. It allows for easy identification of the IOF and is simple to reproduce, especially in countries such as Cameroon where cadaveric dissection is still taboo. Our research was carried out on a fairly large sample of CT-scans and the results give an interesting idea of the general population and how to approach the infraorbital region in surgery. However, our research has a number of limitations such as the fact that measurements on CT-scan can often depend on the position of the cursor and therefore give inadequate measurements. A dry skull study with digital calipers could give results that better reproduce the reality of the IOF morphometry.

## Conclusion

During block anesthesia in a Cameroonian, the practitioner could, after having palpated the vestibular mucosa next to the first molar, follow the line of this tooth to the area where the IOF is located 7 mm from the infraorbital edge and 16 mm from the lateral nasal wall. We have shown that in this sample, the IOF is located medially to the line connecting the wing of the nose to the external palpebral commissure. Thus, during anesthesia by infraorbital block by the extraoral method, the practitioner could insert his needle at the level of the wing of the nose but instead of following the line to the external palpebral commissure, could move more medially 6mm from this virtual line, 16 mm from the lateral nasal wall, 7 mm from the infraorbital border. However, a failure of the infraorbital block can also be explained by the presence of an accessory IOF.

### What is known about this topic

- *The infraorbital foramen is an important structure of the face providing passage to an essential vascular and nervous bundle for the face*;
- *Sectioning this IOF may result in impaired facial sensitivity*.

### What this study adds

- *Our research presents the precise location of this IOF for the surgery of Cameroonian individuals*;
- *Our work is an important tool for the management of V2 essential neuralgia by alcoholization in Cameroonian patients*;
- *Our study on the infraorbital foramen is a pioneer work on the relative position of this structure within the Cameroonian population, this work will provide essential information in facial surgery, particularly in emergency departments where, thanks to our results, the physician will be able to easily locate the IOF and perform infraorbital nerve block anesthesia more easily, thus allowing more effective management of facial wounds*.
